# Classification of Hyperspectral or Trichromatic Measurements of Ocean Color Data into Spectral Classes

**DOI:** 10.3390/s16030413

**Published:** 2016-03-22

**Authors:** Dilip K. Prasad, Krishna Agarwal

**Affiliations:** 1 School of Computer Engineering, Nanyang Technological University, Singapore 639798, Singapore; 2 Singapore-MIT Alliance for Research and Technology, Singapore 138602, Singapore; krishna@smart.mit.edu

**Keywords:** spectral data classification, environmental sensors, ocean color, remote sensing reflectance

## Abstract

We propose a method for classifying radiometric oceanic color data measured by hyperspectral satellite sensors into known spectral classes, irrespective of the downwelling irradiance of the particular day, *i.e.*, the illumination conditions. The focus is not on retrieving the inherent optical properties but to classify the pixels according to the known spectral classes of the reflectances from the ocean. The method compensates for the unknown downwelling irradiance by white balancing the radiometric data at the ocean pixels using the radiometric data of bright pixels (typically from clouds). The white-balanced data is compared with the entries in a pre-calibrated lookup table in which each entry represents the spectral properties of one class. The proposed approach is tested on two datasets of *in situ* measurements and 26 different daylight illumination spectra for medium resolution imaging spectrometer (MERIS), moderate-resolution imaging spectroradiometer (MODIS), sea-viewing wide field-of-view sensor (SeaWiFS), coastal zone color scanner (CZCS), ocean and land colour instrument (OLCI), and visible infrared imaging radiometer suite (VIIRS) sensors. Results are also shown for CIMEL’s SeaPRISM sun photometer sensor used on-board field trips. Accuracy of more than 92% is observed on the validation dataset and more than 86% is observed on the other dataset for all satellite sensors. The potential of applying the algorithms to non-satellite and non-multi-spectral sensors mountable on airborne systems is demonstrated by showing classification results for two consumer cameras. Classification on actual MERIS data is also shown. Additional results comparing the spectra of remote sensing reflectance with level 2 MERIS data and chlorophyll concentration estimates of the data are included.

## 1. Introduction

Satellite hyperspectral sensors are used for remote sensing of terrestrial, oceanic, and atmospheric features. Post-acquisition processing of the hyper-spectral data is important for deriving useful information from the data [1]. One such hyper-spectral processing problem involves ocean color classification [2,3] as an indicator of the health of oceanic environment and biomass [4,5]. For example, it can be useful for monitoring harmful algal blooms. Here, instead of retrieving the inherent optical properties (generally referred to as IOPs) of the constituents of water at the data locations [6], direct classification of the data points into classes characterized by the remote sensing reflectance spectra is sought.

The remote sensing reflectance, denoted as $R_{\mathrm{rs}}$, is the water leaving radiance, *i.e.*, the amplitude and spectral shape of the light reflected by the water surface towards a particular angle when a unit source of flat spectrum (*i.e.*, a perfect source with irradiance 1 $W\cdot m^{-2}\cdot {\mathrm{nm}}^{-1}$) is incident on the surface of water. Thus, it is the ratio of the upwelling radiance from the water column to the downwelling irradiance that enters the water column. On the other hand, the radiometric data measured by a remote sensing satellite sensor, such as MODIS or OLCI is the top-of-atmosphere (TOA) upwelling radiance measured over multiple narrow band channels of the sensor. It contains the upwelling radiance due to atmospheric aerosols, reflection from the ocean’s surface, as well as the upwelling radiance from the ocean’s water column.

For the past several years, satellites have started providing the estimate of $R_{\mathrm{rs}}$ through the calibration of the measured data with extra-terrestrial solar irradiance values and the predictions of atmospheric aerosols properties. The extra-terrestrial solar irradiance values are obtained once every few months in the near-infrared region and interpolated to the visible range channels of the satellite sensors (more details can be found in the MERIS handbook [7]).

Our proposed method alleviates the need for estimating or interpolating different components of upwelling radiances as measured by satellite. It instead uses the concept of white balancing where the radiometric data of clouds is used to compensate indirectly for the downwelling irradiance at the time of measurement of the ocean color data. Furthermore, it stores a lookup table for the radiometric projections of the spectral distributions representing the classes. The white balanced radiometric data is then compared with the entries in the lookup table to identify the spectral class of the data. We note that our method can also be used for classifying the $R_{\mathrm{rs}}$ estimates of the satellites by skipping the process of white balancing. We further show that our approach may enable non-satellite sensors such as the multi-band SeaPRISM sensor and extend the use of the trichromatic consumer cameras for environmental sensing. This indicates potential use of robot-mounted consumer cameras as practical and cost-effective environmental sensors.

We highlight that clustering of *in situ* or sensor estimates of remote sensing reflectance spectra is not the main proposition of the paper. The aim is to assign a known class, which may be obtained by clustering or may correspond to the IOPs of the water column, to the measured data directly. Thus, it is categorically different from [8,9,10] in its aim. A trivial difference is that our classes are defined in the hyperspectral space, thus the same classes can be projected onto any sensor to create a corresponding lookup table. On the other hand, the clustering in [8,9,10] is done on the multispectral space. However, our main proposition is to use cloud pixels or white references for white balancing the multispectral data such that better classification accuracy is achieved:

- without requiring atmospheric correction for satellite sensors, despite the contribution of the remote sensing reflectance being only a small part of the TOA radiances, or
- without requiring mutlispectral sensors, such that airborne broad band sensors such as consumer cameras can be used.

We note that [8,9,10] use the remote sensing reflectance estimates of the satellite data, *i.e.*, level 2 data product of the satellite sensors. On the other hand, the utility of our method is being able to derive direct correspondence between sensor’s actual measurements and the classes of remote sensing reflectance which indicate some useful characteristics of remote sensing reflectance.

## 2. Information about Datasets and Sensors for Synthetic Experiments

Here, we present the details of the datasets and sensors that we use throughout the paper for the synthetic results.

**Figure 1:**
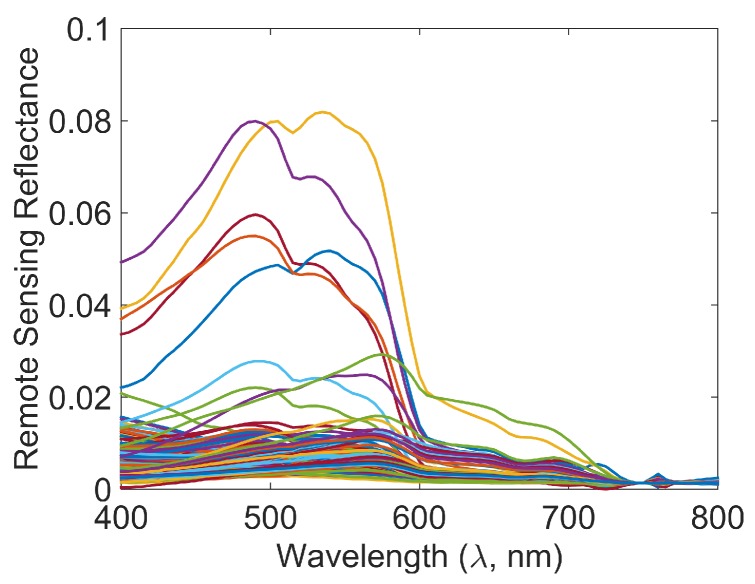
Spectral remote sensing reflectances ${\vec{R}}_{m}$ in Dataset 1.

**Dataset 1:** The authors of [11] have collected the *in situ* remote sensing reflectance spectra of samples collected from all over the world, from the East China Sea in the east to Hawaii in the west, as reported in Table 1 of [11]. The data is spectrally diverse and from sources spanning shallow waters, deep oceans, prolific algal masses, and harmful red algae bloom with distinct red tinge. Further details about the data can be found in [11]. The remote sensing reflectances of the 281 locations from [11] are reproduced in Figure 1. For the ease of reference, we call this dataset Dataset 1.

**Figure 2:**
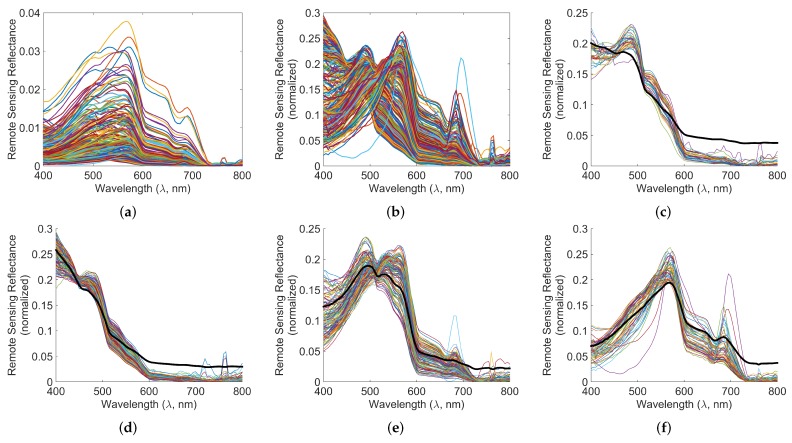
Normalized spectral data and spectral clusters of Dataset 2. The thick black lines in subfigures (**b**–**f**) show the centroids ${\vec{\rho }}^{\circ }$ of the clusters. (**a**) Reflectances ${\vec{R}}_{m}$; (**b**) normalized reflectances ${\vec{\rho }}_{m}$; (**c**) Class 1; (**d**) Class 4; (**e**) Class 6; (**f**) Class 8.

**Dataset 2:** Dataset 2 contains 350 data points collected at several times and locations in the South Florida and West Florida bay. The data has been taken from the oceanic data repository [12]. The remote sensing reflectances of the data points in this dataset are reproduced in Figure 2a.

**Figure 3:**
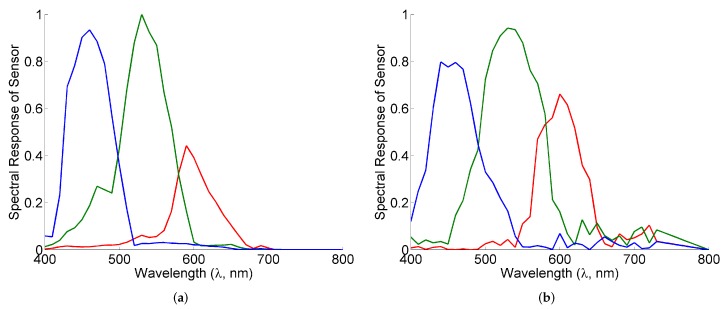
Spectral responses of the various channels of sensors of the consumer camera considered in this paper. (**a**) Canon 1D Mark III; (**b**) Nikon D40.

**Sensors:** Six satellite remote sensors—MODIS (8 bands), MERIS (11 bands), SeaWiFS (7 bands), CZCS (4 bands), OLCI (15 bands), and VIIRS (7 bands)—are used in the synthetic experiments. A field measurement multiband photometer SeaPRISM from CIMEL Electronique has also been included. Their spectral bands are noted in Table 1. Additionally, our paper considers two consumer cameras as examples of unconventional airborne sensors, Canon 1Ds Mark III and Nikon D40, the spectral responses of which are presented in Figure 3.

**Figure 4:**
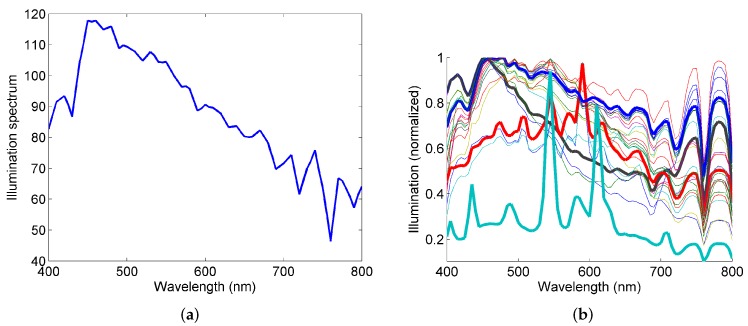
Illumination spectrum of D65 illumination (**a**) and 25 illuminations measured in outdoor scenarios at various locations and times in Barnard dataset (**b**). The thick lines show the general trends of these illuminations in (b).

**Dataset of illumination spectra for downwelling irradiance:** For the synthetic experiments, we use the illumination spectrum of D65 light shown in Figure 4a since it is considered similar to the daylight illumination spectrum [13]. Barnard dataset is a dataset of illumination spectra measured in outdoor conditions on different days and times [14]. These illuminations are shown in Figure 4b and the major indicative trends are shown in thick lines.

## 3. Proposed Classification Approach

In this section, we present the details of the proposed approach. For the ease of following the notations, we have listed them in Table 2. We first layout the foundations for our approach by modelling the radiometric measurement as the projections of remote sensing reflectance spectra and the downwelling irradiance. Next, we discuss the proposed classification approach. We also include an example of forming the lookup table.

### 3.1. Radiometric Measurements as Functions of Remote Sensing Reflectance Spectra

Let the remote sensing reflectance corresponding to *m*th data point (a geographic location) be represented as:

(1) $${\vec{R}}_{m}={\left[r_{m,1},r_{m,2},\ldots ,r_{m,B}\right]}^{T}$$

where $r_{n}$ is the remote sensing reflectance r(λ) corresponding to the wavelength $\lambda_{n}$, the superscript T denotes vector and matrix transpose, and the number of wavelength samples *N* is sufficiently large to represent the spectral reflectances.

Let the sensor, satellite or airborne, have *B* bands (channels) where the *b*th channel is characterised by its spectral sensitivity ${\vec{S}}_{b}$. The spectral response of the sensor be denoted as:

(2) $$S={\left[{\vec{S}}_{1}\;{\vec{S}}_{2}\;\ldots \;{\vec{S}}_{B}\right]}^{T}$$

The measurement at a sensor corresponding to the *m*th data point is given as:

(3) $${\vec{X}}_{m}={\left[x_{m,1},x_{m,2},\ldots ,x_{m,B}\right]}^{T}$$

and is related to the incoming upwelling radiance ${\vec{L}}_{m}={\left[L_{m,1},L_{m,2},\ldots ,L_{m,B}\right]}^{T}$ as:

(4) $${\vec{X}}_{m}=S{\vec{L}}_{m}$$

Below, we describe the upwelling radiances ${\vec{L}}_{m}$ at satellite and airborne sensors as functions of the remote sensing reflectances ${\vec{R}}_{m}$.

#### 3.1.1. Satellite Sensors

Let us denote the total TOA upwelling radiance as L(λ), the upwelling radiance due to the water column as $L_{\mathrm{water}}\left(\lambda \right)$, and the downwelling irradiance as $E_{\mathrm{down}}\left(\lambda \right)$. For convenience, we combine the upwelling radiance due to atmospheric aerosols and reflection from the ocean’s surface and refer to them as atmosphere’s upwelling radiance $L_{\mathrm{atmos}}\left(\lambda \right)$. Thus, TOA upwelling radiance is given as:

(5) $$L\left(\lambda \right)=L_{\mathrm{atmos}}\left(\lambda \right)+t\left(\lambda \right)L_{\mathrm{water}}\left(\lambda \right)$$

and $L_{\mathrm{water}}\left(\lambda \right)$ is given as:

(6) $$L_{\mathrm{water}}\left(\lambda \right)=r\left(\lambda \right)E_{\mathrm{down}}\left(\lambda \right)$$

where t(λ) denotes the transmittance from the water surface to the TOA as a function of wavelength.

For a shadow region in the satellite image, it is showed in [15,16] that $L_{\mathrm{atmos}}\left(\lambda \right)$ can be computed as:

(7) $$L_{\mathrm{atmos}}\left(\lambda \right)=L_{\mathrm{sun}}\left(\lambda \right)-\frac{L_{\mathrm{sun}}\left(\lambda \right)-L_{\mathrm{shd}}\left(\lambda \right)}{1-\frac{E_{\mathrm{down},\mathrm{diffuse}}\left(\lambda \right)}{E_{\mathrm{down}}\left(\lambda \right)}}$$

where $L_{\mathrm{sun}}\left(\lambda \right)$ and $L_{\mathrm{shd}}\left(\lambda \right)$ are the values of L(λ) at close-by regions in sunny and shadowed ocean. $E_{\mathrm{down},\mathrm{diffuse}}\left(\lambda \right)$ is the component of $E_{\mathrm{down}}\left(\lambda \right)$ corresponding to the diffuse downwelling irradiance due to atmospheric scattering. Furthermore, in the cloud and shadow method discussed in [15,16], after deriving $L_{\mathrm{atmos}}\left(\lambda \right)$ using Equation (7), the remote sensing reflectance r(λ) is computed as:

(8) $$r\left(\lambda \right)=r_{\mathrm{cloud}}\frac{L_{\mathrm{sun}}\left(\lambda \right)-L_{\mathrm{atmos}}\left(\lambda \right)}{L_{\mathrm{cloud}}\left(\lambda \right)-L_{\mathrm{atmos}}\left(\lambda \right)}$$

where $r_{\mathrm{cloud}}$ and $L_{\mathrm{cloud}}\left(\lambda \right)$ are the reflectance of the cloud and the TOA upwelling radiance from the cloud to the sensor, respectively. We note that in the visible region, $r_{\mathrm{cloud}}$ is spectrally flat for optically thick clouds [17,18].

**Figure 5:**
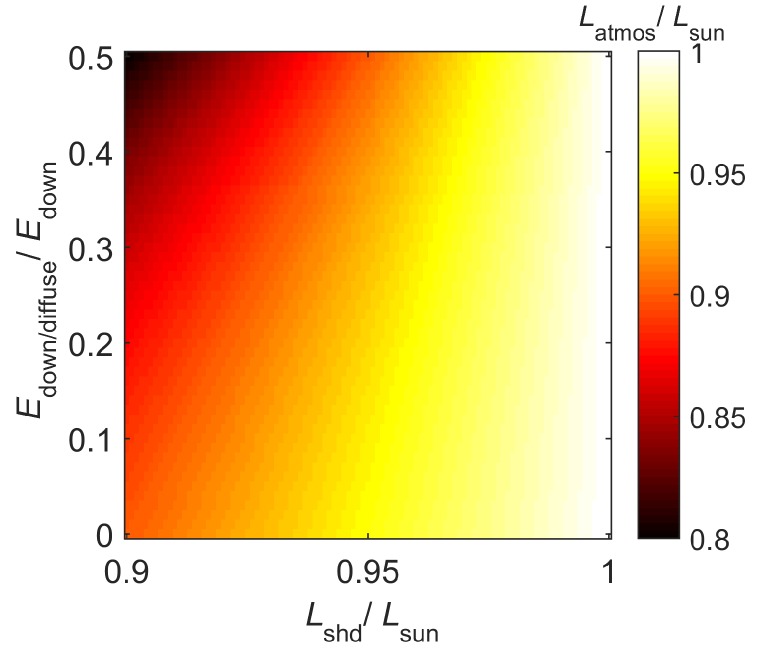
The ratio $\frac{L_{\mathrm{atmos}}}{L_{\mathrm{sun}}}$ is plotted as a function of $\frac{L_{\mathrm{shd}}}{L_{\mathrm{sun}}}$ and $\frac{E_{\mathrm{down},\mathrm{diffuse}}}{E_{\mathrm{down}}}$ for an arbitrary wavelength *λ*.

Here, we derive approximations for Equations (7) and (8) such that the remote sensing reflectance is an approximate expression of the TOA upwelling radiances measured at the ocean in a sunny location and a nearby cloud. It is noted in [15,16] that $L_{\mathrm{atmos}}\left(\lambda \right)$ is relatively less sensitive to the ratio $\frac{E_{\mathrm{down},\mathrm{diffuse}}\left(\lambda \right)}{E_{\mathrm{down}}\left(\lambda \right)}$ because $\frac{L_{\mathrm{shd}}\left(\lambda \right)}{L_{\mathrm{sun}}\left(\lambda \right)}$ is close to 1. In order to verify this, we perform a synthetic experiment for an arbitrary *λ*, where we assign values in the range 0.9 to 1 to $\frac{L_{\mathrm{shd}}}{L_{\mathrm{sun}}}$ and values in the range 0 to 0.5 to $\frac{E_{\mathrm{down},\mathrm{diffuse}}}{E_{\mathrm{down}}}$. For these values, we compute $\frac{L_{\mathrm{atmos}}}{L_{\mathrm{sun}}}$ and plot them in Figure 5. It is seen that, despite significant variation in the ratio $\frac{E_{\mathrm{down},\mathrm{diffuse}}}{E_{\mathrm{down}}}$, the value of the ratio $\frac{L_{\mathrm{atmos}}}{L_{\mathrm{sun}}}$ remains more than 0.8. Thus, practically, $\frac{L_{\mathrm{atmos}}\left(\lambda \right)}{L_{\mathrm{sun}}\left(\lambda \right)}$ can be represented as β(λ), where β(λ) is close to 1. Since β(λ) is close to 1, it can be safely represented as a constant β≈1. Here, we have used β=0.75 in order to allow for potentially more variation in $\frac{E_{\mathrm{down},\mathrm{diffuse}}\left(\lambda \right)}{E_{\mathrm{down}}\left(\lambda \right)}$ and $\frac{L_{\mathrm{atmos}}\left(\lambda \right)}{L_{\mathrm{sun}}\left(\lambda \right)}$ in practical scenario. Later, in Section 4.2.4, we show that the proposed classification algorithm is not too sensitive to the value of *β*. Thus, Equation (8) can be modified as follows:

(9) $$r(\lambda )=\alpha \frac{L_{\mathrm{sun}}\left(\lambda \right)}{L_{\mathrm{cloud}}\left(\lambda \right)-\beta L_{\mathrm{sun}}\left(\lambda \right)}$$

where $\alpha =r_{\mathrm{cloud}}\left(1-\beta \right)$ is a constant and can be suppressed from further consideration. In addition, we suppress the subscript sun. Thus, the radiometric data measurements corresponding to L(λ) and $L_{\mathrm{cloud}}\left(\lambda \right)$ are represented as $\vec{X}$ and ${\vec{X}}_{\mathrm{cloud}}$, respectively.

#### 3.1.2. Airborne Sensors

For an airborne sensor close to the water surface, the measured upwelling radiance is the product of the remote sensing reflectance and the downwelling irradiance, *i.e.*,:

(10) $$L\left(\lambda \right)=r\left(\lambda \right)E_{\mathrm{down}}\left(\lambda \right)$$

As opposed to the use of radiometric data at cloud for satellite sensors, the airborne sensors have the flexibility of using a white patch or other white references including the clouds in the scene, such that the reference white data at the sensor corresponds to:

(11) $$L_{\mathrm{white}}\left(\lambda \right)=r_{\mathrm{white}}E_{\mathrm{down}}\left(\lambda \right)$$

where $r_{\mathrm{white}}$ is the reflectance of the reference patch and is expected to have almost flat spectrum. Consequently, it can be dropped from further consideration as a constant. Combining Equations (10) and (11), we get:

(12) $$r\left(\lambda \right)=\frac{L(\lambda )}{L_{\mathrm{white}}\left(\lambda \right)}$$

### 3.2. Classification Approach

**Figure 6:**
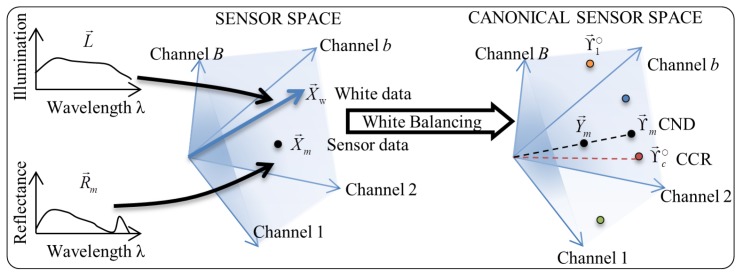
Illustration of the process of classification of the data using the lookup table.

We first describe the canonical space, which is the space of white balanced radiometric data as well as the entries of the lookup table. The lookup table contains the reference radiometric data values for the representative spectra of the spectral classes, which we call as the canonical class representatives (CCRs). This is discussed next. Lastly, we discuss the classification scheme itself. The classification process is illustrated in Figure 6.

**Canonical space for measurements at TOA for satellite sensors:** Suppose that under a given downwelling irradiance $E_{\mathrm{down}}\left(\lambda \right)$, the sensor generates a radiometric data $\vec{X}$ at the data point and the radiometric data ${\vec{X}}_{\mathrm{cloud}}$ at the nearby cloud. See a discussion on the criterion for proximity of the cloud to the data point in Section 4.2.5. Using Equation (9), we define canonical data $\vec{Y}$ as follows:

(13) $$\vec{Y}={\left(X_{w}\right)}^{-1}\vec{X}$$

where $X_{w}=\mathrm{diag}\left({\vec{X}}_{\mathrm{cloud}}-\beta \vec{X}\right)$ and the space of $\vec{Y}$ is called the canonical space. We note that this operation of compensating the data using the white data is called white balancing in the field of image processing [19].

**Canonical space for airborne sensor measurements:** Suppose that under a given illumination $\vec{L}$, an airborne sensor generates radiometric data $\vec{X}$ and white data ${\vec{X}}_{w}$. Then, using Equation (12), we define a canonical data $\vec{Y}$ as follows:

(14) $$\vec{Y}={\left(X_{w}\right)}^{-1}\vec{X}$$

where $X_{w}=\mathrm{diag}\left({\vec{X}}_{w}\right)$ and the space of $\vec{Y}$ is called the canonical space.

**Canonical space for pre-estimated $R_{\mathrm{rs}}$:** If the classification of pre-estimated $R_{\mathrm{rs}}$ is sought instead of the measured radiometric data, the conversion to the canonical space through Equation (14) need not be performed and we note that in this case $\vec{Y}$ is the projection of the $R_{\mathrm{rs}}$ only on the sensor space, *i.e.*, $\vec{Y}=S{\vec{R}}_{m}$.

**Lookup table:** The spectral classes are represented by their normalized reflectance spectra $\left\{{\vec{\rho }}_{c}^{\circ }\right\}$. The normalized reflectance spectra here mean that the L2 norm $∥{\vec{\rho }}_{c}^{\circ }∥$ of each of these vectors is 1, where the L2 norm is defined as the square root of the sum of squares of all the elements of the vector. The lookup table for a sensor contains the canonical radiometric data ${\vec{\Upsilon }}_{c}^{\circ }$ corresponding to the normalized reflectance spectra $\left\{{\vec{\rho }}_{c}^{\circ }\right\}$ representing the spectral classes:

(15) $${\vec{\Upsilon }}_{c}^{\circ }=S{\vec{\rho }}_{c}^{\circ }$$

The CCRs stored in the lookup table are ${\vec{\Upsilon }}_{c}^{\circ }$. An example of the formation of the lookup table is discussed in Section 3.3.

The hyperspectral remote sensing reflectances of the classes are projected onto the sensors through Equation (15). In the context of the sensor, whose data is actually being classified, the projected multispectral class reflectances do not introduce any loss of the spectral information since the information lost in hyperspectral-to-multispectral projection cannot be captured by the sensor anyways.

**Classification of the data:** We normalize the input data transformed to the canonical space $\vec{Y}$ to form the canonical normalized data (CND) ${\vec{\Upsilon }}_{m}$:

(16) $${\vec{\Upsilon }}_{m}=\frac{{\vec{Y}}_{m}}{∥{\vec{Y}}_{m}∥}$$

Due to the normalization, ${\vec{\Upsilon }}_{m}$ is directly comparable to the CCRs ${\vec{\Upsilon }}_{c}^{\circ }$. Now, for classification, we compute the angle between the *m*th CND and the *c*th CCR as follows:

(17) $$\theta_{m,c}=∠\left({\vec{\Upsilon }}_{m},{\vec{\Upsilon }}_{c}^{\circ }\right)$$

where

(18) $$∠\left(\vec{a},\vec{b}\right)=\cos^{-1}\left(\frac{\vec{a}\cdot \vec{b}}{∥\vec{a}∥∥\vec{b}∥}\right)$$

The class $\alpha_{m}$ of the *m*th data is then identified as:

(19) $$\alpha_{m}=\arg\min\left(\theta_{m,c};\forall c\right)$$

### 3.3. An Example of Finding Characteristic $R_{\mathrm{rs}}$ Spectra for the Lookup Table

We present an example for forming the lookup table by finding characteristic $R_{\mathrm{rs}}$ spectra of classes in the lookup table from *in situ* remote sensing reflectance spectra. Additionally, for a data point’s $R_{\mathrm{rs}}$ spectrum, assignment of the class is also discussed. Our lookup table is formed assuming that the $R_{\mathrm{rs}}$ spectra of the classes are known *a priori*. For this paper, we identified such classes by unsupervised clustering of remote sensing reflectance spectra of the Dataset 1, shown in Figure 1, using the popular *k*-means clustering approach [20], which has been found useful in ocean data classification as well [10]. Such an approach is useful when the knowledge of corresponding IOPs is unavailable. During *k*-means clustering, we included the magnitudes of reflectance spectra as a feature in order to separate classes with similar spectra but different amplitudes, potentially correlated to different chlorophyll concentrations. However, such unsupervised clustering may not be needed if IOPs are known.

**Figure 7:**
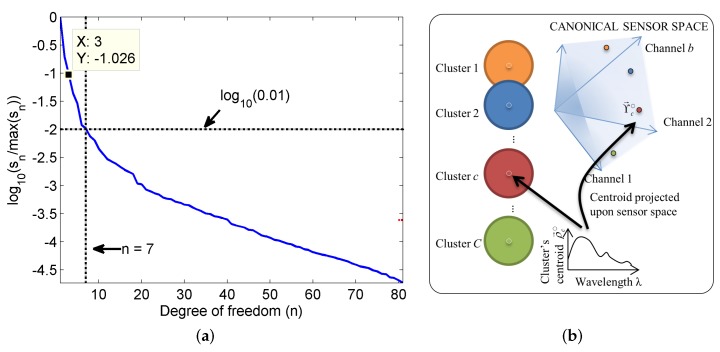
Estimating a suitable value for the number of clusters can be done by analyzing the singular values of the reflectance data (**a**); illustration of the formation of the lookup table in given in (**b**).

**Determining suitable number of clusters:** The *k*-means clustering approach, though unsupervised, requires that the number of classes be specified. An estimate of the number of classes can be obtained by determining the number of linear independent components sufficient to represent the entire dataset well. Here, we use singular value decomposition to obtain this estimate. Singular value decomposition gives the orthogonal independent basis vectors representing the data and the singular values represent the strengths of these independent vectors [21]. Figure 7a shows log values of the normalized singular values $\sigma_{n}/\max\left(\sigma_{n}\right)$, where $\sigma_{n}$ are the singular values obtained using singular value decomposition of the matrix $R=\left[\begin{array}{cccc} {\vec{\rho }}_{1} & {\vec{\rho }}_{2} & \ldots & {\vec{\rho }}_{M} \end{array}\right]$ and ${\vec{\rho }}_{m}$ are the normalized $R_{\mathrm{rs}}$ spectra given by:

(20) ρ→m=R→mR→mR→m∥R→m∥

It is seen that n=8 corresponds to a very small ratio of $\sigma_{n}$/$\max(\sigma_{n})$ (<1%). This indicates that the data can be represented well by 8 independent clusters. Thus, we have used C=8 clusters.

***k*****-means clustering of** ***in situ*** **remote sensing reflectance spectra:** The algorithm of *k*-means clustering aims at clustering *M* data points into *C* clusters in which each member of the cluster is nearest to the centroid (mean) of the cluster. The *c*th cluster forms a class denoted as $C_{c}$ and is characterized by the cluster’s centroid ${\vec{v}}_{c}^{\circ }$.

For clustering, instead of using only the $R_{\mathrm{rs}}$ spectrum ${\vec{R}}_{m}$ as the feature of a data point, we use the normalized spectrum ${\vec{\rho }}_{m}$ and the amplitude of the spectrum $∥{\vec{R}}_{m}∥$ together to form the data feature. Thus, the feature vectors ${\vec{v}}_{m}$ used for clustering are given as:

(21) $${\vec{v}}_{m}=\left[\begin{array}{c} {\vec{\rho }}_{m}\\ ∥{\vec{R}}_{m}∥ \end{array}\right]$$

Taking the normalized spectrum helps in clustering the data which have similar spectra but only different amplitudes, thus using only the spectral shape rather than the amplitude. On the other hand, including the amplitude as an additional attribute helps in retaining at least some information about the amplitude.

After clustering the data, we use the following metric to evaluate the quality of clustering result:

(22) $$E=\sum_{\forall c}\left[d_{c,\min,\mathrm{intercluster}}-d_{c,\mathrm{mean},\mathrm{intracluster}}\right]$$

where $d_{c,\min,\mathrm{intercluster}}$ is the distance of the centroid of the nearest class from the centroid of the *c*th class and $d_{c,\mathrm{mean},\mathrm{intracluster}}$ is the mean distance between the members in the *c*th class. Mathematically, $d_{c,\min,\mathrm{intercluster}}$ and $d_{c,\mathrm{mean},\mathrm{intracluster}}$ are given as:

(23) $$d_{c,\min,\mathrm{intercluster}}=\min\left(\theta_{c,c^{\prime }};\forall c^{\prime }\right)$$

(24) $$\theta_{c,c^{\prime }}=∠\left({\vec{\rho }}_{c}^{\circ },{\vec{\rho }}_{c^{\prime }}^{\circ }\right)$$

(25) $$d_{c,\mathrm{mean},\mathrm{intracluster}}=\mathrm{mean}\left(\theta_{c}\left({\vec{\rho }}_{m}\right);\forall {\vec{\rho }}_{m}\in C_{c}\right)$$

(26) $$\theta_{c}\left({\vec{\rho }}_{m}\right)=\mathrm{mean}\left(∠\left({\vec{\rho }}_{m},{\vec{\rho }}_{m^{\prime }}\right);\forall m^{\prime }\right)\;;{\vec{\rho }}_{m},{\vec{\rho }}_{m^{\prime }}\in C_{c}$$

where ${\vec{\rho }}_{c}^{\circ }$ is the spectral reflectance corresponding to ${\vec{v}}_{c}^{\circ }$. Out of 20 executions (or runs), we choose the clustering results that has the maximum value of *E*.

**Figure 8:**
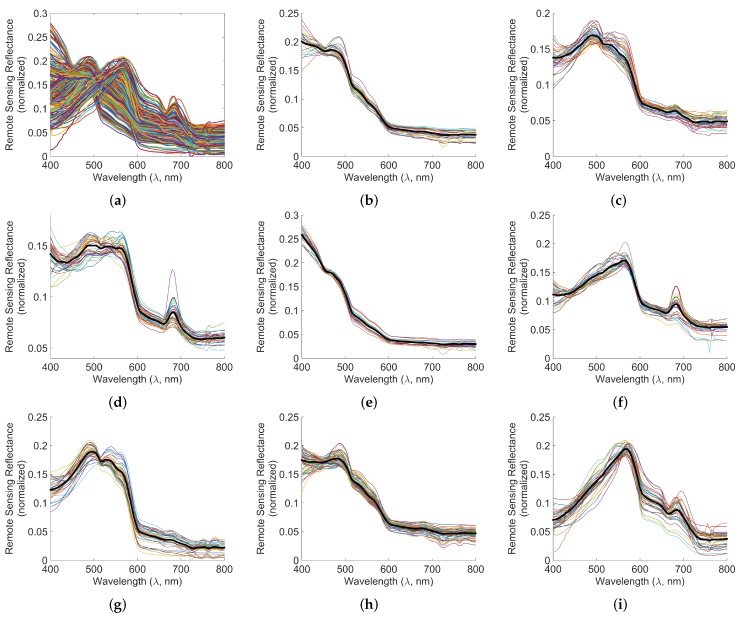
Normalized spectral data and spectral clusters of Dataset 1. The thick black lines in subfigures (**b**–**i**) show the centroids ${\vec{\rho }}^{\circ }$ of the clusters. (**a**) Normalized reflectances ${\vec{\rho }}_{m}$; (**b**) Class 1; (**c**) Class 2; (**d**) Class 3; (**e**) Class 4; (**f**) Class 5; (**g**) Class 6; (**h**) Class 7; (**i**) Class 8.

The normalized remote sensing reflectance spectra of the samples in the Dataset 1 are shown in Figure 8a. The clustering results, *i.e.*, classes are shown in Figure 8b–i. The thick black line shows the centroid ${\vec{\rho }}_{c}^{\circ }$ and the thin lines show the members ${\vec{\rho }}_{m}$ of the class $C_{c}$ in each sub-figure.

**Assigning class to a $R_{\mathrm{rs}}$ spectrum:** The class assignment for an *in situ* remote sensing reflectance $R_{\mathrm{rs}}$ with the data vector ${\vec{R}}_{m}$ can be done as follows:

(27) $$c_{m}=\arg\min\left(∠\left({\vec{\rho }}_{m},{\vec{\rho }}_{c}^{\circ }\right);\forall c\right)$$

This has been used for assigning the classes to the data points as shown in Figure 8b–i for Dataset 1 and Figure 2c–f for Dataset 2. It is interesting that the data in Dataset 2 can be assigned to only four classes, *viz.* Classes 1, 4, 6, 8, as seen in Figure 2.

## 4. Results

### 4.1. Synthetic Experiments

Here, we present four synthetic experiments using two datasets of *in situ* measurements of $R_{\mathrm{rs}}$, the details of which are given in Section 2. For these synthetic experiments, the lookup table has been formed using Dataset 1. Six satellite sensors (see Section 2), MODIS, MERIS, SeaWiFS, CZCS, OLCI, and VIIRS and one field sensor, SeaPRISM, are used. Classification results are shown in Dataset 1 and Dataset 2 assuming D65 illumination (see Section 2), which represent a general daylight illumination pattern (*i.e.*, downwelling irradiance). The robustness of the method to various illumination spectra is also shown. Finally, we show that our classification approach can be used for commercial cameras as well, although with reduced performance.

Classification results are quantified using the precision and recall measures, which are defined as follows:

(28) $$\mathrm{Precision}\left(c\right)=\frac{\mathrm{count}(\alpha_{m}=c_{m})}{\mathrm{count}(\alpha_{m})};\forall \left(c_{m}=c\right)$$

(29) $$\mathrm{Recall}\left(c\right)=\frac{\mathrm{count}(\alpha_{m}=c_{m})}{\mathrm{count}(c_{m})};\forall \left(c_{m}=c\right)$$

We note that the numerators in both precision and recall are the true-positive classifications while the denominators of precision and recall are (true-positive + false positive) and (true-positive + false negative) classifications, respectively. Further, while overall precision cannot be defined for a dataset, overall recall for a dataset is defined as the ratio of total number of correct classifications to the total number of data points in the dataset.

#### 4.1.1. Classification Results for Dataset 1

The classification results in terms of precision and recall measures for Dataset 1 are listed in Table 3 and discussed below. We observe that overall recall for all the satellite and field sensors is more than 90%, and the recall is more than 95% for MODIS, MERIS, OLCI, and VIIRS.

In general, precisions for Classes 1 and 2 are poorer than other classes for any sensor, indicating that data points from other classes may get misclassified as Classes 1 and 2 with much more likelihood than other classes. This is not unexpected since the normalized spectral reflectances of Classes 1 and 2 (black line in Figure 8b,c) also represent the general trends of other classes except their peculiarities.

Another observation is that the proposed algorithm performs well for Class 8 across all the sensors. This is because Class 8 has quite a different spectral pattern in the range 400–600 nm as compared to other classes and a definite “valley and peak” in the range 650–700 nm, as seen in the black line in Figure 8i.

#### 4.1.2. Classification Results for Dataset 2

While Dataset 1 was used for forming the lookup table and serves as the training dataset, we show classification results for an independent dataset Dataset 2 as the test dataset. The process of identifying the classes of the data in Dataset 2 from the *in situ* measurements is presented in Section 3.3. Here, we note that all data in Dataset 2 get classified into one of the four classes only, namely Class 1, 4, 6 and 8.

The classification results of the proposed classification approach using the four satellite sensors are presented in Table 4. The recall values for MODIS, MERIS, OLCI and VIIRS sensors are more than 94%. This is only a slight degradation from the results of the Dataset 1 presented in Table 3.

The performance for SeaWiFS and SeaPRISM sensors degrades significantly, giving a recall of only 86.83% and 80.11%, respectively, for Dataset 2. Its precisions for Classes 1 and 8 are specially quite low. This is because several data points actually belonging to Class 4 get classified as Class 1, and several data points actually belonging to Class 6 get classified as Class 8. Notably, band 470–490 nm is crucial for separating Classes 1 and 4, and this band is missing in both SeaWiFS and SeaPRISM. Similarly, the crucial band 560–580 nm for separating Classes 6 and 8 is also missing in both of these sensors.

The performance for the CZCS sensor is quite good, experiencing a degradation of less than 1% in comparison to the result for Dataset 1 in Table 3. It is noticeable from Table 3 and Table 4 that the proposed method can give good results for commercial cameras as well, which have only three bands each and all the bands are broadband. Thus, while having a large number of bands and good radiometric quality indeed ensures good classification, the key factor in separability of the classes is the distance between the clusters in the sensor space (irrespective of the dimension of sensor space). Here, the relatively broader bands of CZCS as compared to other satellite sensors, allows for gathering contributions from the more distinguishing wavelengths, although the contribution gets smeared by other adjacent wavelengths in the same band. Thus, the loss in number of channels is partially compensated by the larger bandwidth of each channel, thus allowing for good performance even for CZCS.

#### 4.1.3. Different Illuminations and Classification Accuracy

The illumination spectrum of the daylight may change everyday and several times a day depending upon the time (sunrise, noon, *etc.*), cloudiness, rain, *etc*. Thus, it is important to test the performance of the proposed classification method and sensor for data that corresponds to a wide variety of illumination spectra encountered in daylight. We test our method using 25 different illumination spectra from the Barnard dataset [14], the details of which are presented in Section 2.

The overall recall results for different illuminations are computed. The average value of these overall results for each sensor is tabulated in Table 5. It is seen that the values are quite close to the overall results shown for D65 illumination in Table 3 and Table 4. The results clearly demonstrate the robustness of the proposed algorithm in different illumination conditions.

#### 4.1.4. Classification Using Consumer Cameras

In addition to satellite sensors, we also consider if the proposed classification can be used with consumer cameras as well. For this, we consider two consumer cameras, Canon 1Ds Mark III (Canon) and Nikon D40 (Nikon), which have only three channels of significantly wider bandwidth than the satellite sensors. The classification results for Dataset 1, Dataset 2, and different illuminations experiment are presented in Table 3, Table 5 and Table 5, respectively. It is seen that despite having three wide band channels only, these cameras give a recall of more than 85% for the validation Dataset 1 and more than 75% for Dataset 2. Furthermore, the average results for different illuminations are also similar. This indicates the robustness of the proposed method to the choice of sensor and the utility of the proposed method for both satellite and ground based sensors.

### 4.2. Classification of Real Satellite Data

Here, we consider examples of classification of the raw radiometric data actually measured by MERIS. We used full resolution level 1 files which have geolocated and calibrated radiance information and pixel classification labels for identifying ocean pixels, bright pixels, and the other pixels (referred to as unlabelled in the context of our algorithm). In the level 1 data of MERIS, the labels of pixels allow us to select the cloud/bright pixels, ocean pixels, and the unlabelled pixels. According to the labels of level 1 data, the chosen ocean pixels are essentially the valid, non-coastline, unsuspicious, fully-measured, ocean pixels in clear sky with no glint risk (determined by level 1 processing); the chosen bright pixels are essentially the valid, non-coastline, unsuspicious, fully measured, ocean pixels in bright sky with no glint risk (thus cloud). However, the glint labels of level 1 data assume flat ocean surface and thus do not exclude the occurrence of glint due to rough and dynamic ocean surface. We refer the readers to Section 4.2.6 for consideration of glint while using our algorithm. The lookup table of our method uses the reflectances learnt from Dataset 1 as discussed in Section 3.3.

#### 4.2.1. Classification of MERIS Data over Different Scenes

**Figure 9:**
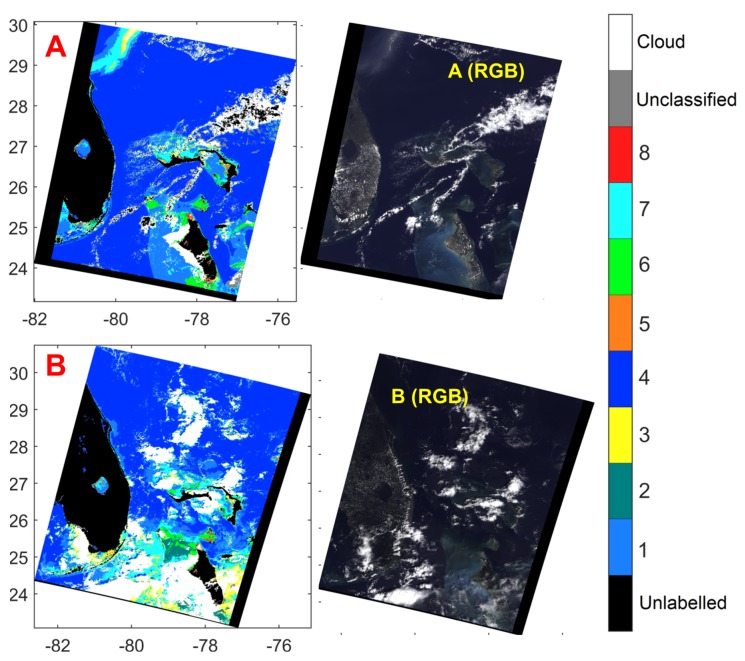
Classification results using MERIS’s raw radiometric data. Data of scene A was taken on 7 May 2011, 15:21 p.m. Data of scene B was taken on 29 September 2005, 15:39 p.m. The Red-Green-Blue (RGB) projections of the three scenes are also shown.

For each data point, the average radiance for each channel for all the bright pixels within 110 km radius is used as the cloud data. Then, each ocean pixel is classified using the proposed algorithm. If the angles between the canonical normalized data at a point $\Upsilon_{m}$ and all the spectral classes $\Upsilon_{c}^{\circ }$ are more than 15 degrees, the pixel is left unclassified. The threshold of 15 degrees incorporates the possibility that the classes may not have represented all the spectra and some spectra may appear similar to one of the classes but not similar enough. The results are shown in Figure 9. Most pixels are assigned to Classes 1, 3, 4, and 7. The other classes are also observed in a small percentage of pixels in scenes A and B. We note that our algorithm does not classify or label certain pixels as unlabelled or cloud. The unlabelled and cloud pixels are determined by the MERIS data labels. The algorithm then classifies only the ocean pixels into classes 1 to 8, or labels them as unclassified.

#### 4.2.2. Correlation with IOP Results in the Same Data

**Figure 10:**
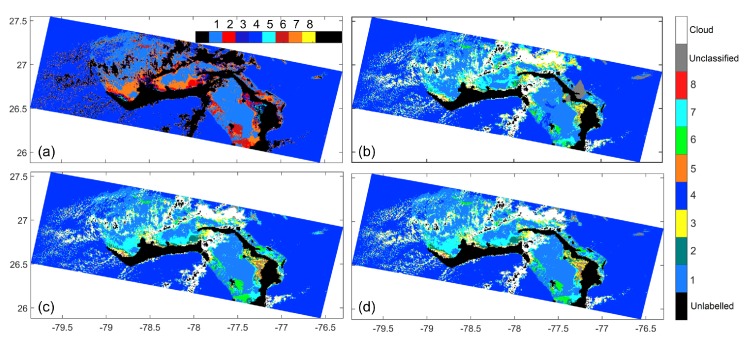
Classification of MERIS data from scene A shown in Figure 9—(**a**) the classification result in Figure 9 but with a different color map; (**b**–**d**) classification using nearest cloud ${\mathrm{Opt}}_{\mathrm{nearest}}$, clouds in proximity ${\mathrm{Opt}}_{\mathrm{proximity}}$, and all clouds ${\mathrm{Opt}}_{\mathrm{all}}$, respectively.

In order to demonstrate some insight into the connection of the spectral classification with the IOPs, we consider the MERIS data of scene A in Figure 9, which was acquired on 7 May 2011 at 15:21 p.m. We particularly focus of the classification results at Grand Bahamas, for which [22] plotted the surface concentrations of chlorophyll “a” in Figure 17 of [22]. In order to visually enhance the correspondence between Figure 17 of [22] and our classification results, the result is plotted with a modified color map in Figure 10a. The Classes 2, 6, and 7 show good correspondence with the high chlorophyll “a” concentrations in Figure 17 of [22].

#### 4.2.3. Similarity between Estimated Remote Sensing Reflectances Using MERIS Level 2 Data and the Proposed Method

Here, we investigate if the normalized canonical data for satellite sensor, obtained through Equation (16), matches spectrally with the remote sensing reflectances estimated in the level 2 product of the MERIS data. For this, we consider the Grand Bahamas region. We plot the cosine similarity (also called the goodness of fit coefficient [13,23,24]) as follows:

(30) $$\gamma \left(\vec{\Upsilon },{\vec{R}}_{\mathrm{MERIS}}\right)=\frac{{\vec{\Upsilon }}^{T}{\vec{R}}_{\mathrm{MERIS}}}{∥\vec{\Upsilon }∥\;\;∥{\vec{R}}_{\mathrm{MERIS}}∥}$$

**Figure 11:**
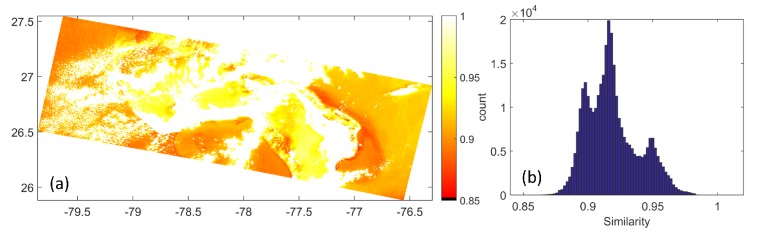
Similarity between the estimates of the remote sensing reflectance spectra from the proposed algorithm and the level 2 MERIS data of the same day and time—(**a**) similarity map (**b**) histogram of the similarity values.

The cosine similarity map for the Grand Bahamas region is shown in Figure 11a, and the histogram of the similarity values is potted in Figure 11b. It is seen that the similarity is very high across all the data points, with maximum of data points demonstrating a similarity of 0.91. The mean similarity for the entire region in Figure 11a is 0.913. This shows good spectral agreement between the remote sensing reflectances estimated using the proposed method and MERIS level 2 processing.

#### 4.2.4. Effect of the Value *β* on Classification

**Figure 12:**
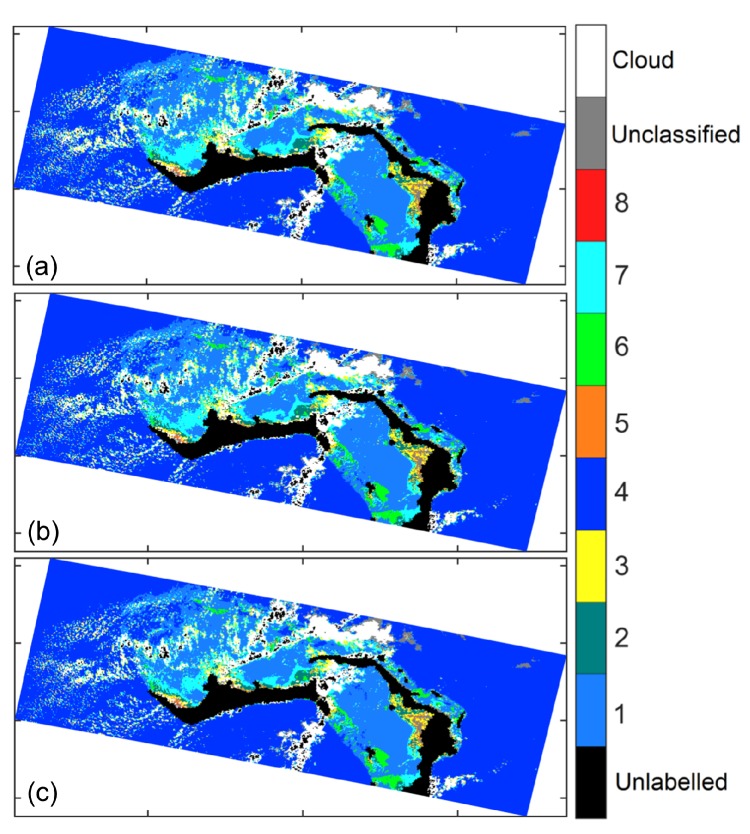
Classification results for Grand Bahamas region using different values of *β*. (**a**) β=0.80; (**b**) β=0.85; (**c**) β=0.90.

As discussed in Section 3.1.1, although the value of $\frac{L_{\mathrm{atmos}}\left(\lambda \right)}{L_{\mathrm{sun}}\left(\lambda \right)}$ depends upon the ratios $\frac{L_{\mathrm{shd}}\left(\lambda \right)}{L_{\mathrm{sun}}\left(\lambda \right)}$ and $\frac{E_{\mathrm{down},\mathrm{diffuse}}\left(\lambda \right)}{E_{\mathrm{down}}\left(\lambda \right)}$, since the ratio $\frac{L_{\mathrm{atmos}}\left(\lambda \right)}{L_{\mathrm{sun}}\left(\lambda \right)}$ does not change much for the practically valid condition $\frac{L_{\mathrm{shd}}\left(\lambda \right)}{L_{\mathrm{sun}}\left(\lambda \right)}\approx 1$. Thus, the ratio $\frac{L_{\mathrm{atmos}}\left(\lambda \right)}{L_{\mathrm{sun}}\left(\lambda \right)}$ was assigned a constant value β≈1. We mentioned that we have used β=0.75. Here, we show that the classification results are relatively not sensitive to the value of *β*. Considering three more values of *β*, β=0.80,0.85,0.90, we plot the classification results for the Grand Bahamas region in Figure 12. We also tabulate the percentage of pixels classified differently in Table 6. It is noted that classification results do not vary much with the value of *β*.

#### 4.2.5. Assumption of Local Uniformity

An underlying assumption in our formulation for both satellite and airborne sensors is that the downwelling irradiance and general atmospheric conditions such as aerosol concentrations and earth surface reflections are locally uniform in the region containing the data point and the cloud or the white reference. Here, we discuss the criterion for calling the downwelling irradiance as locally uniform. Indeed, the downwelling irradiance is a function of the zenith angle, the local cloud cover, local weather conditions, the azimuthal angle, *etc.*, which may be different over any two points, besides the constant factors such as the time of the day and the earth’s position on its orbit. Notably, the trigonometric and other terms which do not vary with the wavelength do not affect the proposed algorithm because of the normalization of the canonical data as explained in Equation (16).

For the other spectral quantities, it can be assumed that the conditions do not vary significantly over say few 10 s of kilometers from the perspective of the above defined variables. We note that the locally uniform assumption here should also be related to the sensor position. For example, an airborne sensor at a height of several meters will see the local uniformity of the conditions differently than an airborne sensor at a height of a few kilometers, which, in turn, will see local uniformity differently than a satellite sensor. Thus, in addition to restricting the distance between the data point and the white reference to a few tens of kilometers, it is also important to consider the planar angle *θ* subtended by the distance between the data point and the white reference on the sensor. If the planar angle *θ* is sufficiently small, the geometric projections of the data point on the white reference given by cos(θ) are close to 1. Suppose that the tolerable difference in the projections is 1%. Accordingly, for $\theta \leq 8^{\circ }$, the conditions may be considered uniform. For a satellite sensor such as MERIS and OLCI, which are at a distance of approximately 800 km from the earth, the distance between the data point and the cloud, neglecting the difference in altitude, is about 110 km. On the other hand, for an airborne sensor at a height of 80 m from the earth’s surface, the distance is 11 m.

However, using the example of the Grand Bahamas shown in Figure 10b–d, we show that our approach is relatively robust against the choice of the cloud region. We consider three options for choosing the cloud region. The first option, ${\mathrm{Opt}}_{\mathrm{nearest}}$, is to use only the radiometric data corresponding to the cloud closest to the data point. This result is shown in Figure 10b. The second option, ${\mathrm{Opt}}_{\mathrm{proximity}}$, as used for generating the results in Figure 9, is the mean data corresponding to the all the cloud pixels within 110 km of the data point. The result is shown in Figure 10c. The last option, ${\mathrm{Opt}}_{\mathrm{all}}$, is the mean data corresponding to all the pixels in the scene portion of Grand Bahamas shown in Figure 10a. The maximum distance between cloud pixel and data point in the scene is about 390 km. The classification result for this option are shown in Figure 10d. We tabulate the percentage of pixels classified differently between any two options in Table 7. When the unclassified pixels are included in computing, the percentage values are determined as pixels classified differently in the two options out of the total number of ocean pixels. When the unclassified pixels are excluded, the percentage values are determined as pixels assigned a valid class but classified differently in the two options out of the total number of ocean pixels that are assigned a valid class in both of the options. The results in Figure 10b–d show that the ${\mathrm{Opt}}_{\mathrm{proximity}}$ and ${\mathrm{Opt}}_{\mathrm{all}}$ options are more robust, resulting in fewer unclassified pixels. Table 7 further shows that using different options results only in small variations in the classification.

We note that for satellite sensors, there may be better approaches to determine the distance of local uniformity, such as through the estimates of aerosol optical depth across a region. However, such approaches mandate the use of either processed satellite data (such as level 2 data) or other algorithms for estimating the aerosol depth, which may also introduce further approximations. Therefore, we consider that the above mentioned approximation may suffice.

#### 4.2.6. Consideration of Glint

We note that our algorithm does not apply for the cases where glint is present. The reason is that the radiance from glint can exceed the radiance from both the ocean and atmosphere and the radiance from glint is not considered in the proposed algorithm. Sensors such as MERIS do not avoid glint. For example, although the level 1 data of MERIS does label pixels with high glint, advanced processing not assuming a flat ocean surface is needed to identify pixels with low and medium glint risk [7,25]. Thus, in practice, glint has to be computed using methods such as [26]. Then, either glint can be compensated computationally or the pixels with glint can be identified and excluded before applying our algorithm. A simple but approximate way of excluding the possibility of glint is to consider only those pixels for which the angular difference between the solar specular vector (from the sun to the measurement point) and the view vector (from the sensor to the measurement point) is more than 30${}^{\circ }$ such that the radiance due to glint has a small contribution to the measurement.

## 5. Conclusions

In this paper, a classification method is proposed for classifying satellite data into user-specified spectral classes corresponding to the remote sensing reflectances of water samples. The method can be used with both the raw radiometric data of the satellites as well as the estimated remote sensing reflectance spectra derived from satellites’ post-processing modules. When using the raw data, the method uses raw data from clouds to compensate for the unknown downwelling irradiance.

Synthetic results show good recall and precision for satellite sensors, giving better than 85% recall for two datasets of *in situ* measurements. Robustness against diverse downwelling irradiance spectra is also verified. As an extension, we show good classification results for two consumer cameras as well, despite them having only three channels of significantly wide bandwidth. Examples of classifications using actual MERIS level 1 data are also shown. Several studies are conducted to demonstrate the robustness of the proposed approach to different parameters. An example of analogy between IOP results and our classification results is also given. In addition, the $R_{\mathrm{rs}}$ predicted using MERIS data modules corresponds well with the intermediate normalized spectra computed in our approach.

As future work, it is interesting to design lookup tables in which the CCRs correspond to specific IOPs of the water samples. The requirement is that the spectra corresponding to distinct IOPs should be spectrally separable. Then, instead of classifying the water samples, the inverse problem of reconstructing the IOPs may be cast as determining (potentially linear) combinations of CCRs.

## Figures and Tables

**Table 1:** Information of the bands of satellite sensors.

	MODIS	MERIS	SeaWiFS	CZCS	OLCI	VIIRS (VisNIR)	CIMEL SeaPRISM

No. of bands	9	11	8	4	21	9	9
No. used (*B*)	8	11	7	4	15	7	6
Bands	405–420	405.2–419.6	402–422	425–460	407.5–417.5	402–422	407–417
used (nm)	438–448	435.0–449.5	433–453	500–535	437.5–447.5	436–454	435–445
	483–493	482.3–496.9	480–500	535–565	485–495	478–498	495–505
	526–536	502.2–516.8	500–520	650–685	505–515	545–565	526–536
	546–556	552.1–566.7	545–565		555–565	600–680	545–555
	662–672	612.0–626.6	660–680		615–625	662–682	670–680
	673–683	657.0–671.6	745–785		660–670	739–754	
	743–753	674.5–686.6			670–677.5		
		700.7–715.3			677.5–685		
		747.0–759.0			703.75–713.75		
		755.8–764.1			750–757.5		
					760–762.5		
					762.5–766.25		
					766.25–768.75		
					771.25–786.25		
Bands	862–877		845–885		392.5–407.5	846–885 (I2)	
unused (nm)					855–875	846–885 (M7)	865–875
					880–890		931–941
					895–905		1015–1025
					930–950		
					1000–1040		

**Table 2:** Useful notations for our method and their meaning are presented here.

Symbol	Meaning
*n*; *N*	index of the wavelength sample $\lambda_{n}$; total number of wavelength samples
*m*; *M*	index of the location of measurement; total number of location samples
*c*; *C*	index of the spectral class; total number of spectral classes
*b*; *B*	index of the channel in a sensor; total number of channels in a sensor
L(λ)	Upwelling radiance measured at the sensor
$L_{\mathrm{water}}\left(\lambda \right)$	Upwelling radiance leaving the water column
$L_{\mathrm{atmos}}\left(\lambda \right)$	Upwelling radiance at radiance due to atmospheric scattering and reflection from water surface
$L_{\mathrm{sun}}\left(\lambda \right)$, $L_{\mathrm{shd}}\left(\lambda \right)$, $L_{\mathrm{cloud}}\left(\lambda \right)$	Upwelling radiance at sensor at sunny, shadowed, and cloud regions, respectively
$E_{\mathrm{down}}\left(\lambda \right)$	Downwelling radiance at the water column
$E_{\mathrm{down},\mathrm{diffuse}}\left(\lambda \right)$	Portion of $E_{\mathrm{down}}\left(\lambda \right)$ corresponding to atmospheric scattering
r(λ); $r_{n}$	remote sensing reflectance at wavelength *λ*; $r_{n}=r\left(\lambda_{n}\right)$
${\vec{R}}_{m}$	remote sensing reflectance at the *m*th location Equation (1)
${\vec{\rho }}_{m}$	normalized remote sensing reflectance at the *m*th location Equation (20)
${\vec{\rho }}_{c}^{\circ }$	normalized remote sensing reflectance representing the *c*th spectral class
$r_{\mathrm{cloud}}$	Spectrally flat remote sensing reflectance of cloud
β(λ); *β*	Ratio $\frac{L_{\mathrm{atmos}}\left(\lambda \right)}{L_{\mathrm{sun}}\left(\lambda \right)}$; constant approximation of β(λ)
*α*; $\alpha_{m}$	Constant $\alpha =r_{\mathrm{cloud}}\beta$; class assigned to *m*th data point by our algorithm using Equation (19)
S	sensor’s spectral response matrix given as $\left[{\vec{S}}_{1}{\vec{S}}_{2},\ldots {\vec{S}}_{B}\right]$
${\vec{S}}_{b}$	spectral sensitivity of the *b*th channel of sensor given as ${\left[s_{\left(b,1\right)},s_{\left(b,2\right)},\ldots ,s_{\left(b,N\right)}\right]}^{T}$
$\vec{X}$	sensor’s radiometric measurement (data) given as ${\left[x_{1},x_{2},\ldots ,x_{B}\right]}^{T}$
${\vec{X}}_{w}$	sensor’s white data computed differently for satellite and airborne sensors
${\vec{\Upsilon }}_{c}^{\circ }$	canonical class representative (CCR) of the *c*th class stored in the lookup table
${\vec{Y}}_{m}$	canonical data obtained using data ${\vec{X}}_{m}$ and ${\vec{X}}_{w}$ in Equation (14)
${\vec{\Upsilon }}_{m}$	canonical normalized data (CND) computed using Equation (16)

**Table 3:** Classification results using the proposed algorithm for Dataset 1.

Sensor	Measure	Class 1	Class 2	Class 3	Class 4	Class 5	Class 6	Class 7	Class 8	Overall
MODIS	Precision	0.8889	0.8810	1.0000	0.9545	0.9667	0.9706	0.9636	0.9744	−
	Recall	0.9231	0.9487	0.9143	1.0000	0.9667	1.0000	0.8983	1.0000	0.9502
MERIS	Precision	0.9630	0.9286	1.0000	1.0000	0.9655	0.9706	1.0000	0.9737	−
	Recall	1.0000	1.0000	0.9429	1.0000	0.9333	1.0000	0.9661	0.9737	0.9751
SeaWiFS	Precision	0.8214	0.8372	1.0000	0.9545	0.9375	0.9412	0.9434	1.0000	−
	Recall	0.8846	0.9231	0.8857	1.0000	1.0000	0.9697	0.8475	1.0000	0.9288
CZCS	Precision	0.8065	0.8372	1.0000	1.0000	0.9355	0.9394	0.9455	0.9744	−
	Recall	0.9615	0.9231	0.8857	0.8571	0.9667	0.9394	0.8814	1.0000	0.9253
OLCI	Precision	0.8667	0.8864	1.0000	1.0000	0.9333	0.9706	1.0000	0.9730	−
	Recall	1.0000	1.0000	0.9143	0.9048	0.9333	1.0000	0.9322	0.9474	0.9502
VIIRS	Precision	0.9259	0.9750	1.0000	0.9545	0.9375	1.0000	1.0000	1.0000	−
	Recall	0.9615	1.0000	0.9429	1.0000	1.0000	1.0000	0.9661	0.9737	0.9786
SeaPRISM	Precision	0.7931	0.8205	1.0000	0.9545	0.9355	0.8421	0.9245	0.9737	−
	Recall	0.8846	0.8205	0.8857	1.0000	0.9667	0.9697	0.8305	0.9737	0.9039
Canon	Precision	0.8889	0.6667	0.6923	1.0000	0.7895	0.9167	0.9483	1.0000	−
	Recall	0.9231	0.7179	0.7714	0.8571	1.0000	0.6667	0.9322	0.9211	0.8505
Nikon	Precision	0.8929	0.7632	0.7027	1.0000	0.7692	0.9310	0.9655	1.0000	−
	Recall	0.9615	0.7436	0.7429	0.8571	1.0000	0.8182	0.9492	0.8947	0.8719

**Table 4:** Classification results using the proposed algorithm for Dataset 2.

Sensor	Measure	Class 1	Class 4	Class 6	Class 8	Overall
MODIS	Precision	0.8000	1.0000	1.0000	0.8438	−
	Recall	1.0000	0.9507	0.9040	1.0000	0.9468
MERIS	Precision	1.0000	1.0000	0.8986	0.9773	−
	Recall	0.9167	1.0000	0.9920	0.7963	0.9580
SeaWiFS	Precision	0.6122	1.0000	0.9904	0.7297	−
	Recall	0.8333	0.8662	0.8240	1.0000	0.8683
CZCS	Precision	0.9286	0.9929	1.0000	0.7941	−
	Recall	0.7222	0.9859	0.8640	1.0000	0.9188
OLCI	Precision	1.0000	0.9861	0.8052	0.9737	−
	Recall	0.5833	1.0000	0.9920	0.6852	0.9468
VIIRS	Precision	0.8182	1.0000	1.0000	0.9000	−
	Recall	1.0000	0.9648	0.9280	1.0000	0.9608
SeaPRISM	Precision	0.5294	1.0000	1.0000	0.6341	−
	Recall	0.5000	0.8873	0.7200	0.9630	0.8011
Canon 1Ds	Precision	0.8065	0.9342	1.0000	1.0000	−
MarkIII	Recall	0.6944	1.0000	0.5520	0.6852	0.7647
Nikon D40	Precision	0.7576	0.9281	1.0000	1.0000	−
	Recall	0.6944	1.0000	0.6000	0.6852	0.7815

**Table 5:** The average overall recall results for the 25 different illuminations are shown here for the different sensors.

Dataset	MODIS	MERIS	SeaWiFS	CZCS	OLCI	VIIRS	SeaPRISM	Canon	Nikon
Dataset 1	0.9502	0.9749	0.9307	0.9267	0.9537	0.9751	0.9004	0.8424	0.8596
Dataset 2	0.9447	0.9581	0.8681	0.9175	0.9107	0.9580	0.8011	0.7622	0.7692

**Table 6:** Percentage of pixels that are classified differently when different values of *β* are used. The pixels that belong to land, cloud, or glint are excluded.

*β*	0.80	0.85	0.9
0.75	2.88	5.97	9.30
0.80	0	3.10	6.45
0.85	3.10	0	3.37

**Table 7:** Percentage of pixels classified differently when different options are used for representing the cloud.

	${\mathrm{Opt}}_{\mathrm{nearest}}$,${\mathrm{Opt}}_{\mathrm{proximity}}$	${\mathrm{Opt}}_{\mathrm{nearest}}$,${\mathrm{Opt}}_{\mathrm{all}}$	${\mathrm{Opt}}_{\mathrm{proximity}}$,${\mathrm{Opt}}_{\mathrm{all}}$
Including unclassified pixels	15.08	14.72	4.52
Excluding unclassified pixels	13.06	12.87	4.06

## References

[1] Zabalza J., Ren J., Zheng J., Han J., Zhao H., Li S., Marshall S. (2015). Novel two-dimensional singular spectrum analysis for effective feature extraction and data classification in hyperspectral imaging. IEEE Trans. Geosci. Remote Sens..

[2] Vantrepotte V., Loisel H., Dessailly D., Mériaux X. (2012). Optical classification of contrasted coastal waters. Remote Sen. Environ..

[3] Mélin F., Vantrepotte V. (2015). How optically diverse is the coastal ocean?. Remote Sens. Environ..

[4] Blondeau-Patissier D., Gower J., Dekker A., Phinn S., Brando V. (2014). A review of ocean color remote sensing methods and statistical techniques for the detection, mapping and analysis of phytoplankton blooms in coastal and open oceans. Prog. Oceanogr..

[5] Cannizzaro J.P., Carder K.L., Chen F.R., Heil C.A., Vargo G.A. (2008). A novel technique for detection of the toxic dinoflagellate, Karenia brevis, in the Gulf of Mexico from remotely sensed ocean color data. Cont. Shelf Res..

[6] Camps-Valls G., Bruzzone L., Rojo-Álvarez J., Melgani F. (2006). Robust support vector regression for biophysical variable estimation from remotely sensed images. IEEE Geosci. Remote Sens. Lett..

[7] ESA (2002). MERIS Product Handbook.

[8] Moore T.S., Campbell J.W., Dowell M.D. (2009). A class-based approach to characterizing and mapping the uncertainty of the MODIS ocean chlorophyll product. Remote Sens. Environ..

[9] Vantrepotte V., Loisel H., Dessailly D., Mériaux X. (2012). Optical classification of contrasted coastal waters. Remote Sens. Environ..

[10] Mélin F., Vantrepotte V. (2015). How optically diverse is the coastal ocean?. Remote Sens. Environ..

[11] Lee Z., Carder K., Arnone R., He M. (2007). Determination of primary spectral bands for remote sensing of aquatic environments. Sensors.

[12] SeaBASS SeaBASS Data Archive Directory. http://seabass.gsfc.nasa.gov/seabasscgi/archive.cgi?q=/USF.

[13] Nguyen R., Prasad D., Brown M., Fleet D., Pajdla T., Schiele B., Tuytelaars T. (2014). Training-Based Spectral Reconstruction from a Single RGB Image. ECCV 2014.

[14] Barnard K., Martin L., Funt B., Coath A. (2002). A data set for color research. Color Res. Appl..

[15] Amin R., Lewis D., Gould R.W., Hou W., Lawson A., Ondrusek M., Arnone R. (2014). Assessing the Application of Cloud-Shadow Atmospheric Correction Algorithm on HICO. IEEE Trans. Geosci. Remote Sens..

[16] Lee Z., Casey B., Arnone R., Weidemann A., Parsons R., Montes M.J., Gao B.C., Goode W., Davis C., Dye J. (2007). Water and bottom properties of a coastal environment derived from Hyperion data measured from the EO-1 spacecraft platform. J. Appl. Remote Sens..

[17] Jedlovec G. (2009). Automated Detection of Clouds in Satellite Imagery.

[18] Chang C., Salinas S., Liew S., Kwoh L. Spectral reflectance of clouds in multiple-resolution satellite remote sensing images. Proceedings of the 27th Asian Conference on Remote Sensing (ACRS).

[19] Cheng D., Prasad D.K., Brown M.S. (2014). Illuminant estimation for color constancy: Why spatial-domain methods work and the role of the color distribution. J. Opt. Soc. Am. A.

[20] MacQueen J. Some methods for classification and analysis of multivariate observations. Proceedings of the fifth Berkeley Symposium on Mathematical Statistics and Probability.

[21] Golub G., Kahan W. (1965). Calculating the singular values and pseudo-inverse of a matrix. J. Soc. Ind. Appl. Math. Ser. B Numer. Anal..

[22] Hu C., Lee Z., Franz B. (2012). Chlorophyll aalgorithms for oligotrophic oceans: A novel approach based on three-band reflectance difference. J. Geophys. Res. Oceans.

[23] Prasad D.K., Wenhe L. (2015). Metrics and statistics of frequency of occurrence of metamerism in consumer cameras for natural scenes. J. Opt. Soc. Am. A.

[24] Prasad D.K. (2015). Gamut expansion of consumer camera to the CIE XYZ color gamut using a specifically designed fourth sensor channel. Appl. Opt..

[25] Cox C., Munk W. (1954). Measurement of the roughness of the sea surface from photographs of the sun’s glitter. JOSA.

[26] Kay S., Hedley J.D., Lavender S. (2009). Sun glint correction of high and low spatial resolution images of aquatic scenes: A review of methods for visible and near-infrared wavelengths. Remote Sens..

[27] Software and Source Codes. https://sites.google.com/site/dilipprasad/source-codes.

